# Diagnostic and prognostic value of the optic nerve sheath diameter with respect to the intracranial pressure and neurological outcome of patients following hemicraniectomy

**DOI:** 10.1186/s12883-018-1202-5

**Published:** 2018-12-05

**Authors:** Yuzhi Gao, Qiang Li, Chunshuang Wu, Shaoyun Liu, Mao Zhang

**Affiliations:** Department of Emergency Medicine, Second Affiliated Hospital, Zhejiang University School of Medicine, No. 88, Jiefang Road, Hangzhou City, 310009 Zhejiang Province China

**Keywords:** Optical nerve sheath diameter, Intracranial pressure, Hemicraniectomy, Neurological outcome

## Abstract

**Background:**

In cases showing cerebrospinal fluid (CSF) redistribution as a compensatory mechanism in acute intracranial hypertension, the optic nerve sheath diameter (ONSD) can be used to estimate intracranial pressure (ICP). However, it remains unclear whether the ONSD can be applied in patients with skull defects after a craniectomy, because the primary injury or surgical craniectomy may alter the dynamics of the CSF circulation or structure of the optical nerve sheath. This study explored the value of the ONSD in patients after a hemicraniectomy.

**Methods:**

This prospective observational study enrolled patients after a hemicraniectomy. All patients underwent invasive ICP monitoring and ocular ultrasound within 6 h postoperatively. We followed the patients for 6 months and evaluated them using the Glasgow Outcome Score (GOS), classifying the outcome as favorable (GOS 4–5) or unfavorable (GOS 1–3). We evaluated the ONSD in both according to the ICP and neurological outcome.

**Results:**

Of the 33 enrolled patients, 20 (60.6%) had an unfavorable outcome at 6 months. Disagreement was seen in the ONSD measurements between the eyes [craniectomy side (ONSDips) and opposite side (ONSDcon)]. The intraclass correlation coefficient between ONSDips and ONSDcon was 0.745 (*p* < 0.001). ONSD had no significant correlation with ICP in Spearman correlation analysis (ONSDips *r* = 0.205, *p* = 0.252; ONSDcon *r* = 0.164, *p* = 0.362). Receiver operator characteristic (ROC) curve analysis revealed that the GCS, Helsinki computed tomography (CT) score, pupil reaction, and ONSDcon measured after the craniectomy were significantly associated with a poor outcome. ONSDcon > 5.5 mm predicted a poor outcome, with an area under the ROC curve of 0.717 (95% confidence interval, 0.534–0.860, *p* = 0.02), 70% sensitivity, and 69.2% specificity.

**Conclusions:**

After hemicraniectomy, the ONSD measured on ultrasound was unreliable for evaluating ICP, but showed potential prognostic value for a poor neurological outcome.

## Background

The optic nerve sheath diameter (ONSD) is often taken as a proxy of intracranial hypertension in brain injury patients [1–3]. The space surrounding the optic nerve is a continuation of the intracranial subarachnoid space. With the compensatory redistribution of cerebrospinal fluid (CSF) seen in cases of intracranial hypertension, the raised intracranial pressure (ICP) instantaneously distends the ONSD [4]. In traumatic brain injury (TBI) and post-cardiac arrest patients, the ONSD calculated based on ultrasound or computed tomography (CT) image is correlated with the invasive ICP [5, 6]. An increase in ONSD from the baseline CT was associated with an unfavorable neurological outcome [7–9]. Although measurement of ONSD by portable ultrasound is feasible and convenient, there are still obstacles to its widespread clinical application [5, 10]. Moreover, some studies found no correlation between ONSD and ICP [11, 12]. Primary brain injury or decompression craniectomy (DC) may alter CSF hydrodynamics or destroy the optic nerve sheath [13, 14]. Therefore, it remains unclear whether ONSD can be applied in patients with skull defects after DC. This study explored the value of the ONSD calculated based on ultrasound images in patients with skull defects following hemicraniectomy.

## Methods

### Setting and participants

This observational study was conducted in a 16-bed intensive care unit (ICU) affiliated with an academic hospital in eastern China. The research protocol was approved by the second Hospital affiliated Zhejiang University Institutional Review Board prior to the start of recruitment and data collection. Almost all of the enrolled neurocritical patients had no ability to express themselves at early stage. So after admitted to our unit, the doctor had a talk with the patients’ immediate family members (spouse, children, parents, et ac) and then they signed an informed consent about patient-related medical treatment. We screened all patients who underwent invasive ICP monitoring after a hemicraniectomy. Exclusion criteria were age < 18 years, ONSD measurement unavailable within 6 h postoperatively, ocular trauma or pre-existing ocular disease, and unsuitable optic nerve sheath images.

### Study protocol

To avoid operator differences, one experienced doctor (WC) performed the ocular ultrasound for every eligible patient and then numbered and stored the ONSD images. Blinded to the ONSD measurements, another doctor (LQ) was in charge of collecting the relevant data from the ocular ultrasound examinations. Before collecting the data, we ensured that the ICP monitors had been zeroed. We followed the patients for 6 months after the injury and evaluated them using the Glasgow Outcome Score (GOS). We classified the patient outcomes as unfavorable (GOS 1, dead; GOS 2, vegetative state; GOS 3, severe disability) and favorable (GOS 4, moderate disability; GOS 5, return to normal life). Two experienced doctors (LS and GY) rechecked the ONSD images and recorded the data. Instead of averaging the values for the two eyes, we classified the ONSD measurements from both eyes into ipsilateral ONSD (craniectomy side, ONSDips) and contralateral ONSD (the side opposite the craniectomy, ONSDcon) according to the side of the craniectomy. Then, we analyzed the ONSD measurements according to the ICP and outcome.

### ONSD measurement

**Fig. 1:**
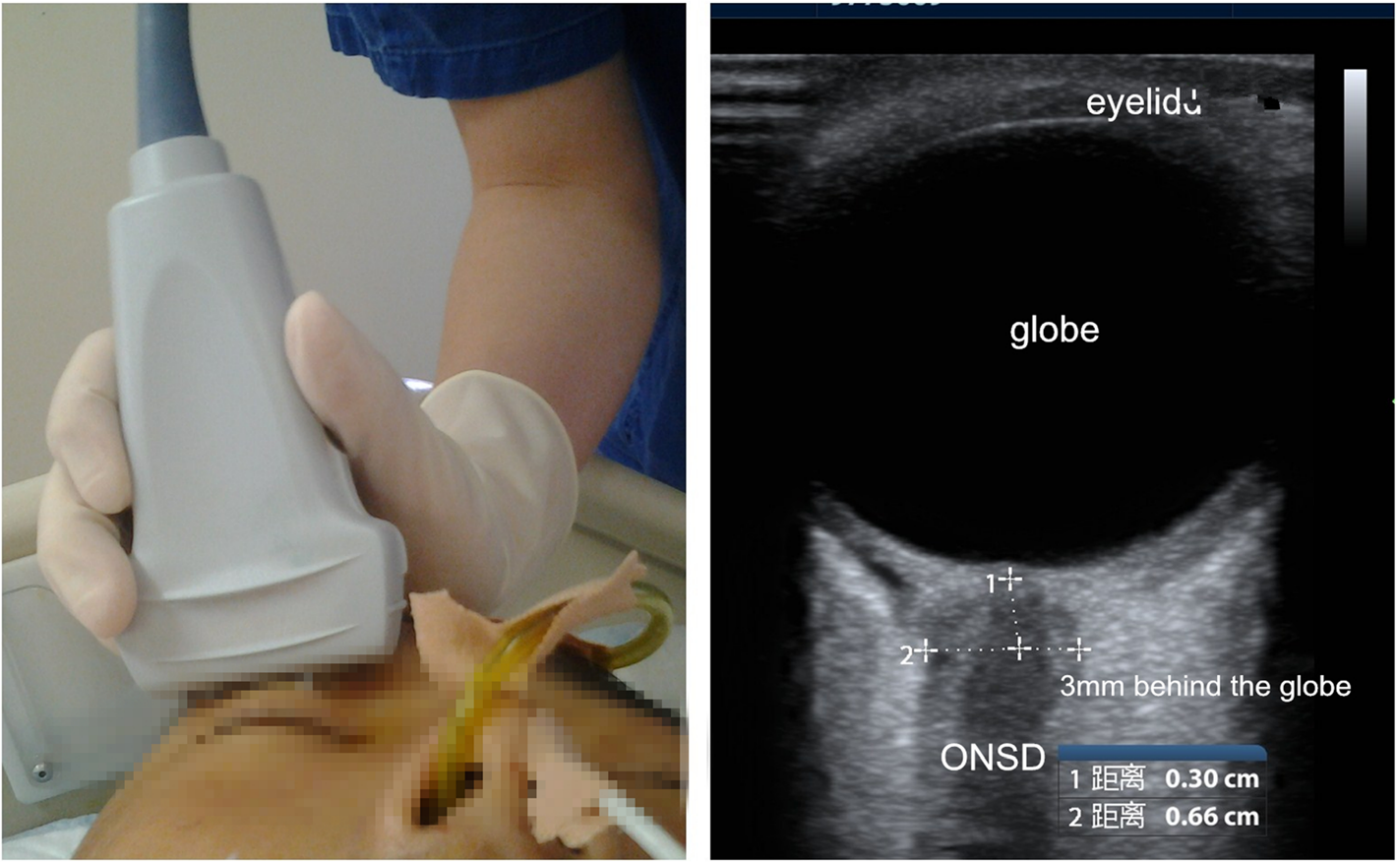
Illustration of ONSD performance and B-mode image of ONSD measurement. A linear high frequency probe placed on the closed eye in transverse visual axis plane, and then adjusted the depth and angle of ultrasound to show the image of ONSD clearly, measure the width of ONSD at 3 mm behind the globe. ONSD optical nerve sheath diameter

A portable ultrasound machine (M9, Mindray, Shenzhen, China) with a linear array probe (13–6 MHz) was used. As described by Blehar et al. [15], we determined ONSD in the visual axis by placing the probe over the closed eyelid. The probe was moved slightly until the optic nerve appeared as a linear hypoechoic object with defined margins behind the globe. After freezing the screen image, we determined ONSD manually 3 mm behind the globe with mechanical calipers (Fig. 1).

### Management of neurocritical patients

All postoperative neurocritical patients received standard intensive care according to the guidelines for management of TBI or intracranial hemorrhage. Ventilation was used to maintain normal oxygenation (PaO_2_ 80–120 mmHg, PaCO_2_ 35–45 mmHg). Midazolam, fentanyl, or sufentanil was infused continuously for sedation and analgesia. Hyperosmolar therapy with 20% mannitol or 3% hypertonic saline was applied when the ICP exceeded 20 mmHg. The cerebral perfusion pressure was maintained at 60~70 mmHg with fluids and norepinephrine as needed. Hypothermia treatment, neuromuscular blocking drugs, and barbiturates were used if indicated clinically.

### Statistics analysis

Variables with normal distributions are expressed as means ± SD, and those with non-normal distributions as medians with interquartile range (IQR). Categorical variables are expressed as n (%). We used Student’s *t-*test or the Kolmogorov–Smirnov test to compare baseline characteristics between patients with favorable and unfavorable outcomes. We constructed Bland–Altman plots and used intraclass correlation coefficient (ICC) analysis to determine the agreement between ONSDips and ONSDcon. Spearman rank correlation was used to evaluate the association of both ONSD measurements with ICP. Receiver operator characteristic (ROC) curves were used to analyze predictors of an unfavorable outcome at 6 months (GOS 1–3). The cutoff values of predictors of a poor outcome (GOS 1–3) were determined using ROC curves. Then, we calculated the sensitivity, specificity, positive predictive value (PPV), and negative predictive value (NPV) for each cutoff. Statistical analyses were performed using MedCalc for Windows software (ver. 11.4; MedCalc Software). *P*-values < 0.05 were considered to be statistically significant.

## Results

### Baseline characteristics

**Fig. 2:**
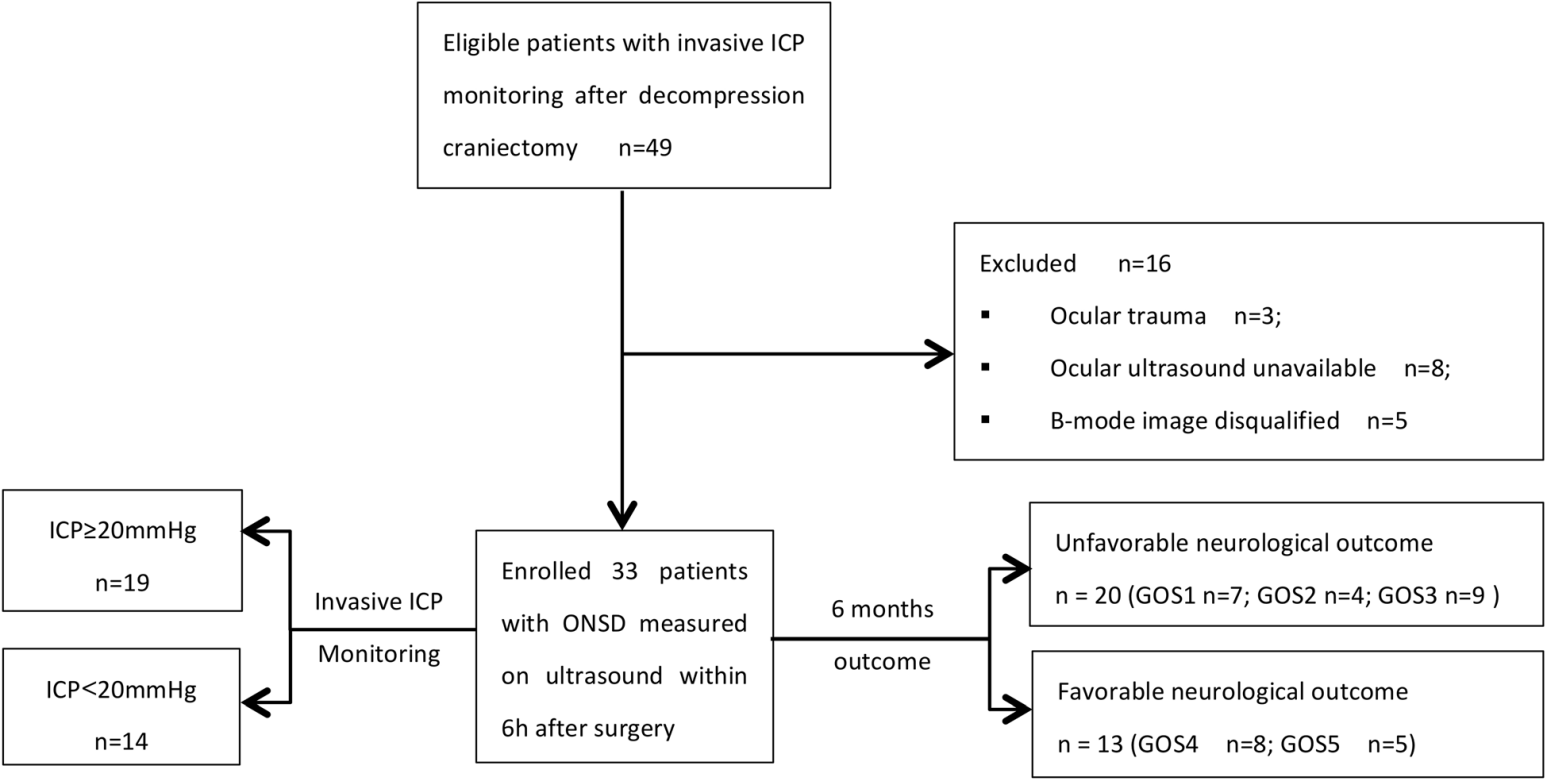
Flowchart of the included patients. ICP intracranial pressure; ONSD optical nerve sheath diameter; GOS Glasgow outcome score

During the study period, there were 49 potentially eligible patients, 16 of whom were excluded because ocular ultrasound was unavailable (*n* = 8), they had ocular trauma (*n* = 3), or the ONSD image was inadequate (*n* = 5). Figure 2 shows a flowchart of the included patients. Of the 33 enrolled patients, 31 (93.9%) had TBI. The outcome at 6 months was favorable (GOS 4–5) in 13 patients (39.4%). Table 1 summarizes the baseline characteristics according to the 6-month neurological outcome. Univariate analysis revealed that the baseline Helsinki CT score, GCS, pupil light reflex before surgery, and ONSDcon after craniectomy were associated with an unfavorable neurological outcome. We found no significant differences in sex, age, reason for surgery, ICP, or ICU stay duration between the patients with unfavorable and favorable outcomes.

**Table 1:** Baseline characteristics

		Neurological outcome (6 months)		
	All	Unfavorable GOS 1–3	Favorable GOS4–5	*p*-value
Number of patients	33	20 (60.6%)	13 (39.4%)	–
Age, y	46.6 ± 14.9	47.9 ± 15.0	46.5 ± 14.1	0.552
Sex (male), n (%)	25 (75.8%)	15 (45.5%)	10 (30.3%)	0.619
Causes of surgery, n (%)				0.474
Traumatic brain injury	31 (93.9%)	14 (42.4%)	17 (51.5%)	–
Hemorrhage stroke	2 (6.1%)	2 (6.1%)	0	–
Initial Glasgow coma score (GCS)				0.008
3–8	22 (66.7%)	17 (51.5%)	5 (15.2%)	–
≥ 9	11 (33.3%)	3 (9.1%)	8 (24.2%)	
Initial Pupil light reflex (n %)				0.039
Both react	20 (60.6%)	9 (27.3%)	11 (33.3%)	
1 reacts	3 (9.1%)	2 (6.1%)	1 (3.0%)	
None reacts	10 (30.3%)	9 (27.3%)	1 (3.0%)	
Helsinki CT score	6 ± 3	7 ± 2	4 ± 3	0.013
ICU duration (day)	8 (6–12)	9 (8–13)	7 (3–12)	0. 079
Hospital discharge (day)	19 ± 9	17 ± 8	22 ± 10	0.134
Data during ocular ultrasound performed within 6 h after craniectomy				
ICP (mmHg)	22 (9–27)	26 (24–32)	14 (9–16)	0.668
MAP (mmHg)	89 (81–97)	89 (82–97)	86 (80–96)	0.337
CPP (mmHg)	68 ± 18	69 ± 20	67 ± 16	0.812
Ipsilateral ONSD (mm)	5.9 ± 0.7	6.0 ± 0.7	5.8 ± 0.5	0.164
Contralateral ONSD (mm)	5.8 ± 0.7	6.1 ± 0.7	5.5 ± 0.7	0.018

*CT* computerized tomography, *ICU* intensive care unit, *ICP* intracranial pressure, *MAP* mean arterial pressure, *CPP* cerebral perfusion pressure, *ONSD* optic nerve sheath diameter, *GOS* glasgow outcome score, GOS 1 dead, GOS 2 vegetative state, GOS 3 severe disability, GOS 4 moderate disability, GOS 5 back to normal life with or without mild disability

**Fig. 3:**
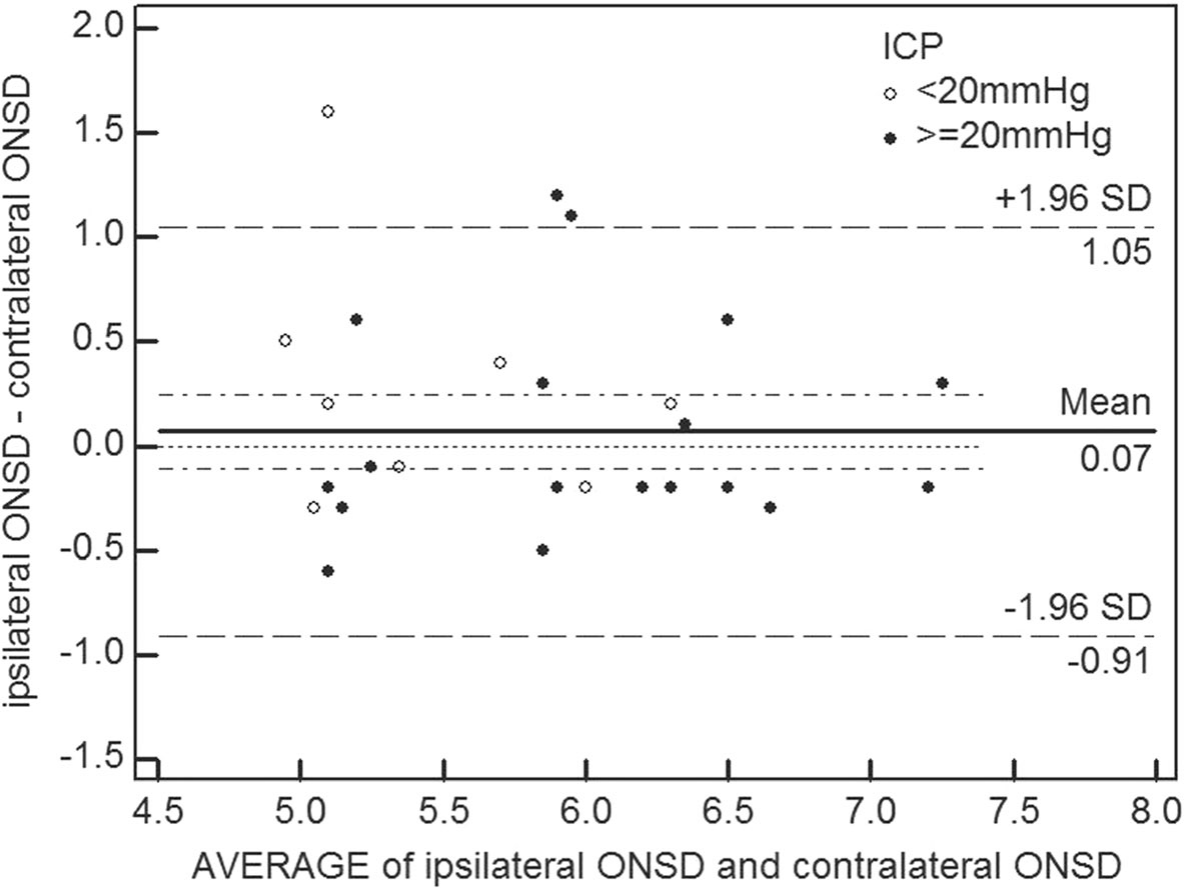
Bilateral ONSD measurements agreement and difference. Bland-Altman plots display the agreement of ipsilateral ONSD and contralateral ONSD measured 3 mm behind the globe by ocular ultrasound. Continuous lines depict the mean of differences; dashed lines denote limits of agreement (mean ± 1.96 times of standard deviation)

In total, 33 paired ONSD measurements of both eyes were obtained from the patients within 6 h after hemicraniectomy. Bland–Altman analysis yielded a mean difference in the bilateral ONSD measurements of 0.07 mm. The limits of agreement were − 0.91 and 1.05 mm, and 9.1% (3/33) of the plots were outside the limits of agreement (Fig. 3). The interclass correlation coefficient of the two ONSD measurements was 0.745 (95% confidence interval [CI]: 0.574–0.867, *p* < 0.001).

### ONSD vs. ICP

**Fig. 4:**
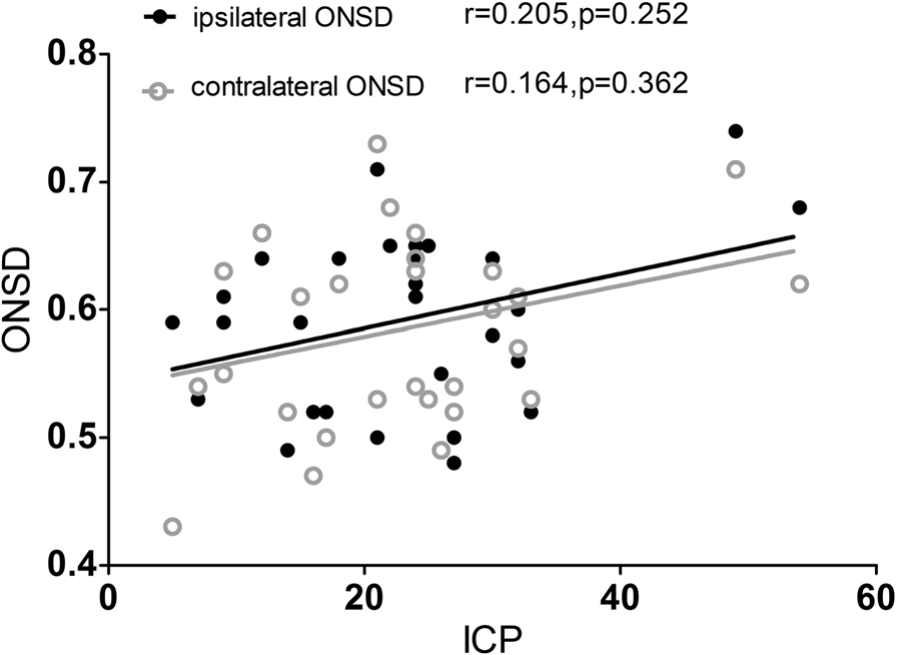
Scatter diagrams of bilateral ONSDs and ICP. There was no significant correlation between bilateral ONSDs and ICP respectively (*p* > 0.05). ONSD optical nerve sheath diameter; ICP intracranial pressure

The ICP had a non-normal distribution, with a median (IQR) of 22 (9–27) mmHg. We applied nonparametric Spearman rank correlation to analyze the relationship between ONSD and ICP, and found no significant correlation (ipsilateral *r* = 0.205, *p* = 0.252; contralateral *r* = 0.164, *p* = 0.362) (Fig. 4). ROC curves for ICP ≥ 20 mmHg were drawn for the ONSD measurements of both eyes. The ONSD of both eyes did not predict a raised ICP (ipsilateral: area under the curve [AUC] = 0.686, 95% CI: 0.502–0.836, *p* = 0.05; contralateral: AUC = 0.677, 95% CI: 0.492–0.828, *p* = 0.66).

### ONSD vs. outcome

ROC curve analysis revealed that the cut-off value of ONSDcon for predicting an unfavorable outcome was 5.5 mm (AUC = 0.717; 95% CI: 0.534–0.860, *p* = 0.02). The sensitivity and specificity of this cut-off value were 70 and 69.2%, respectively. The PPV of a poor outcome was 100% when ONSD > 6.6 mm. The cutoff value of the Helsinki CT score for predicting a poor outcome was 5 (AUC = 0.831, 95% CI: 0.660–0.938, *p* < 0.001), and the sensitivity and specificity were 85 and 69.2%, respectively (see Table 2). On ROC curve analysis, there was no significant difference between the ONSDcon and Helsinki CT score in terms of predicting a poor outcome (*p* = 0.301).

**Table 2:** Parameters in prediction of six months unfavorable outcome (GOS 1–3)

	Cutoff	AUC	95% CI	Sens/Spec(%)	PPV/NPV(%)	*p*-value
Unfavorable outcome (GOS 1–3)						
Ipsilateral ONSD (mm)	6.2	0.642	0.457–0.801	45/84.6	81.8/50	0.15
	6.5			15/100	100/43.3	
Contralateral ONSD (mm)	5.5	0.717	0.534–0.860	70/69.2	73.7/57.1	0.02
	6.6			20/100	100/44.8	
ICP (mmHg)	17	0.638	0.453–0.798	70/53.9	70/53.8	0.17
	33			10/100	100/41.9	
Helsinki CT score	5	0.831	0.660–0.938	85/69.2	81/75	< 0.001
	*7*			*45/100*	*100/54.2*	

*ONSD* optic nerve sheath diameter, *ICP* intracranial pressure, *GOS* glasgow outcome score, *AUC* area under curve, *95% CI* 95% confidence intervals, *Sens* sensitivity, *Spec* specificity, *PPV* positive prediction value, *NPV* negative prediction value

## Discussion

Our data revealed that ONSD was not reliable for noninvasive evaluation of ICP in patients after a hemicraniectomy. First, the ONSD calculated based on postoperative ultrasound images had no correlation with ICP. Second, the diagnostic value of ONSD with respect to a raised ICP (≥ 20 mmHg) was variable in patients after craniectomy. A few studies have reported a weak correlation between ONSD and ICP [11, 12]. Several factors might explain our results; first, the effect of DC complications, such as subdural hygroma and post-traumatic hydrocephalus, on CSF hydrodynamics has been neglected [13]. Surgical craniectomy could reduce the secretion of CSF, affect its outflow, or cause a redistribution of CSF; these altered CSF hydrodynamics might prevent the ONSD from shrinking after the intracranial hypertension relieved by DC [14]. Damage to the optic nerve sheath might also delay or prevent the reversal of ONSD distension [11]. In addition, the standard range of ONSD is inconsistent among studies where a study including a large sample of healthy Chinese adults gave a reference range of ONSD of 4.7–5.4 mm [16].

In TBI and post-cardiac arrest patients, drugs and hypothermia therapy often influence the early prognosis. A few studies have explored the prognostic value of ONSD, calculated based on the initial CT, with respect to the outcome in TBI and post-cardiac arrest patients [7, 8, 17]. In our post-hemicraniectomy patients, increased ONSD was associated with unfavorable outcomes (GOS 1–3). The ONSD reflects the severity of the primary brain injury induced by the initial increase in the ICP, where primary brain injury plays an important role in the neurological prognosis [18]. Although the ONSD was not correlated with the ICP, ONSDcon was significantly greater in patients with unfavorable outcomes (6.1 ± 0.7 vs. 5.5 ± 0.7 mm, *p* = 0.018). The ROC curve analysis indicated that ONSDcon has potential value for predicting an unfavorable outcome. The cutoff value of ONSDcon was 5.5 mm (AUC = 0.717, *p* = 0.02), with a PPV of 100% when ONSDcon > 6.6 mm. As Rajajee et al. surmised, the relationship between ICP and ONSD is contingent on a close temporal association [11]. In our study, primary brain injury and craniectomy itself altered the dynamics of the CSF circulation and structure of the optical nerve sheath, which may have delayed the reversal of ONSD distension after DC. Increased ONSD measured after a craniectomy with a normal or low ICP indicates either severe primary injury or altered CSF hydrodynamics. Such post-craniectomy patients tend to have a poor prognosis.

The bilateral difference in ONSD measurements was another unexpected finding. We classified the ONSD measurements into ONSDips and ONSDcon according to the side of the craniectomy, rather than the right and left side. The patients who underwent hemicraniectomy had non-diffuse primary brain injuries, where higher pressure and greater ONSD might occur on the side with more severe injury [12]. Therefore, we analyzed ONSDips and ONSDcon separately, rather than using the average, including only the larger value in the analyses, as done in previous studies.

Some errors may have influenced our results, for example in terms of the reliability of the ONSD measurements [5, 10]. The ONSD can be measured in the transverse or sagittal plane. Blehar et al. showed that different ONSD measurement methods yielded different values, where more shadowing artifacts arising from the optic disc were observed in the visual axis plane [15]. Therefore, in our study, a single experienced physician performed the ocular ultrasound and used the visual axis transverse plane for the ONSD measurements. We also rechecked the ultrasound images to ensure that unsuitable images were excluded, to avoid measurement errors.

## Conclusions

ONSD was not a reliable index in the noninvasive evaluation of ICP in patients after hemicraniectomy. Due to the influence of the primary brain injury or the craniectomy itself, damage to the optic nerve sheath delayed the reversal of ONSD distention, while the altered CSF hydrodynamics prevented CSF redistribution as a compensatory mechanism in acute intracranial hypertension. Ultrasound measurement of ONSD has potential prognostic value for a poor outcome in patients after hemicraniectomy. An increased ONSD induced by initial intracranial hypertension before DC can reflect the severity of the primary brain injury, which plays an important role in the neurological prognosis.

## Abbreviations

CSF: Cerebral spinal fluid
DC: Decompression craniectomy
GOS: Glasgow outcome score
ICP: Intracranial pressure
NPV: Negative prediction value
ONSD: Optical nerve sheath diameter
PPV: Positive prediction value
TBI: Traumatic brain injury

## References

[1] Rajajee V, Vanaman M, Fletcher JJ, Jacobs TL (2011). Optic nerve ultrasound for the detection of raised intracranial pressure. Neurocrit Care.

[2] Geeraerts T, Launey Y, Martin L, Pottecher J, Vigué B, Duranteau J, Benhamou D (2007). Ultrasonography of the optic nerve sheath may be useful for detecting raised intracranial pressure after severe brain injury. Intens Care Med.

[3] Geeraerts T, Merceron S, Benhamou D, Vigué B, Duranteau J (2008). Non-invasive assessment of intracranial pressure using ocular sonography in neurocritical care patients. Intens Care Med.

[4] Robba C, Bacigaluppi S, Cardim D, Donnelly J, Bertuccio A, Czosnyka M (2016). Non-invasive assessment of intracranial pressure. Acta Neurol Scand.

[5] Dubourg J, Javouhey E, Geeraerts T, Messerer M, Kassai B (2011). Ultrasonography of optic nerve sheath diameter for detection of raised intracranial pressure: a systematic review and meta-analysis. Intens Care Med.

[6] Ohle R, McIsaac SM, Woo MY, Perry JJ (2015). Sonography of the optic nerve sheath diameter for detection of raised intracranial pressure compared to computed tomography: a systematic review and meta-analysis. J Ultrasound Med.

[7] Chelly J, Deye N, Guichard JP, Vodovar D, Vong L, Jochmans S, Thieulot-Rolin N, Sy O, Serbource-Goguel J, Vinsonneau C (2016). The optic nerve sheath diameter as a useful tool for early prediction of outcome after cardiac arrest: a prospective pilot study. RESUSCITATION.

[8] Sekhon MS, McBeth P, Zou J, Qiao L, Kolmodin L, Henderson WR, Reynolds S, Griesdale DEG (2014). Association between optic nerve sheath diameter and mortality in patients with severe traumatic brain injury. Neurocrit Care.

[9] Hwan Kim Y, Ho Lee J, Kun Hong C, Won Cho K, Hoon Yeo J, Ju Kang M, Weon Kim Y, Yul Lee K, Joo Kim J, Youn Hwang S (2014). Feasibility of optic nerve sheath diameter measured on initial brain computed tomography as an early neurologic outcome predictor after cardiac arrest. Acad Emerg Med.

[10] MORETTI R, PIZZI B (2011). Ultrasonography of the optic nerve in neurocritically ill patients. ACTA ANAESTH SCAND.

[11] Rajajee V, Fletcher JJ, Rochlen LR, Jacobs TL (2012). Comparison of accuracy of optic nerve ultrasound for the detection of intracranial hypertension in the setting of acutely fluctuating vs stable intracranial pressure: post-hoc analysis of data from a prospective, blinded single center study. Crit Care.

[12] Strumwasser A, Kwan RO, Yeung L, Miraflor E, Ereso A, Castro-Moure F, Patel A, Sadjadi J, Victorino GP (2011). Sonographic optic nerve sheath diameter as an estimate of intracranial pressure in adult trauma. J Surg Res.

[13] Czosnyka M, Copeman J, Czosnyka Z, McConnell R, Dickinson C, Pickard JD (2000). Post-traumatic hydrocephalus: influence of craniectomy on the CSF circulation. J Neurol Neurosurg Psychiatry.

[14] Shapiro K, Fried A, Takei F, Kohn I (1985). Effect of the skull and dura on neural axis pressure-volume relationships and CSF hydrodynamics. J Neurosurg.

[15] Blehar DJ, Gaspari RJ, Montoya A, Calderon R (2008). Correlation of visual axis and coronal axis measurements of the optic nerve sheath diameter. J Ultrasound Med.

[16] Chen H, Ding GS, Zhao YC, Yu RG, Zhou JX (2015). Ultrasound measurement of optic nerve diameter and optic nerve sheath diameter in healthy Chinese adults. BMC Neurol.

[17] Chae MK, Ko E, Lee JH, Lee TR, Yoon H, Hwang SY, Cha WC, Shin TG, Sim MS, Jo IJ (2016). Better prognostic value with combined optic nerve sheath diameter and grey-to-white matter ratio on initial brain computed tomography in post-cardiac arrest patients. RESUSCITATION.

[18] Soldatos T, Karakitsos D, Chatzimichail K, Papathanasiou M, Gouliamos A, Karabinis A (2008). Optic nerve sonography in the diagnostic evaluation of adult brain injury. Crit Care.

